# Primary extranodal marginal zone non-hodgkin lymphoma of the prostate: a case report

**DOI:** 10.3389/fonc.2026.1721390

**Published:** 2026-03-02

**Authors:** Xiaoru Gao, Xinru Wang, Xiangfei Wang, Hongyi Cai

**Affiliations:** 1 The First Clinical Medicine College of Gansu University of Traditional Chinese Medicine, Lanzhou, China; 2 Department of Radiotherapy I, Gansu Provincial People’s Hospital, Lanzhou, China; 3 Department of Radiotherapy II, Gansu Provincial People’s Hospital, Lanzhou, China; 4 Department of Oncologic Surgery, Gansu Provincial People’s Hospital, Lanzhou, China

**Keywords:** case report, extra-nodal marginal zone-B lymphoma, non-hodgkin lymphoma, prostate, radiation therapy

## Abstract

This report describes a clinically rare case of early-stage primary extranodal marginal zone B-cell lymphoma of the prostate. A 73-year-old male presented with urinary hesitancy. Physical examination revealed mild prostatic enlargement. Prostate biopsy, immunohistochemistry, and genetic testing strongly suggested mucosa-associated lymphoid tissue (MALT) lymphoma. Bone marrow aspiration indicated no bone marrow involvement. Positron Emission Tomography-Computed Tomography (PET-CT) and Emission Computed Tomography (ECT) scans showed no evidence of involvement in other sites. Ultimately, the patient was definitively diagnosed with primary prostatic MALT lymphoma, Ann Arbor stage IE. He underwent external beam radiation therapy to the prostate, receiving a total dose of 3000 cGy delivered in 15 fractions. During radiotherapy, the patient experienced no significant adverse reactions. Following treatment, urinary obstruction symptoms markedly improved. Follow-up abdominal and urinary tract ultrasound examinations at 1week, 1month, and 4months post-radiotherapy showed marked tumor regression with no signs of recurrence. This report aims to share the treatment experience of this case, hoping to provide insights for clinicians.

## 1 Introduction

Primary extranodal marginal zone B-cell lymphoma, also known as MALT lymphoma, is a relatively uncommon subtype of non-Hodgkin lymphoma (NHL). Its exclusive involvement of the prostate is exceptionally rare. Clinically, patients with this condition often present initially with urinary symptoms. In the early stages, they frequently lack the classic “B symptoms” of lymphoma (fever, night sweats, weight loss) and lymphadenopathy, making it easy to confuse with common urological conditions such as benign prostatic hyperplasia or prostatitis. Currently, no standardized diagnostic and therapeutic protocols exist for prostate lymphoma. This report details a 73-year-old male patient with primary prostate MALT lymphoma whose initial presentation was urinary hesitancy. The treatment approach selected was radical radiotherapy, aiming to provide clinical reference for similar rare cases.

## 2 Patient information

A 73-year-old male patient presented to our hospital on March 10, 2025, presenting with a 1-week history of dysuria, urinary dribbling, frequency, and urgency. The patient reported no systemic B symptoms such as fever, weight loss, or night sweats. Upon admission, symptomatic treatment with mirabegron sustained-release tablets resulted in significant improvement. Physical examination revealed mild prostatic enlargement without palpable lymphadenopathy or hepatosplenomegaly. The patient had a generally good health status, with a history of hypertension and diabetes mellitus. He had no history of smoking or alcohol consumption.

**Figure 1:**
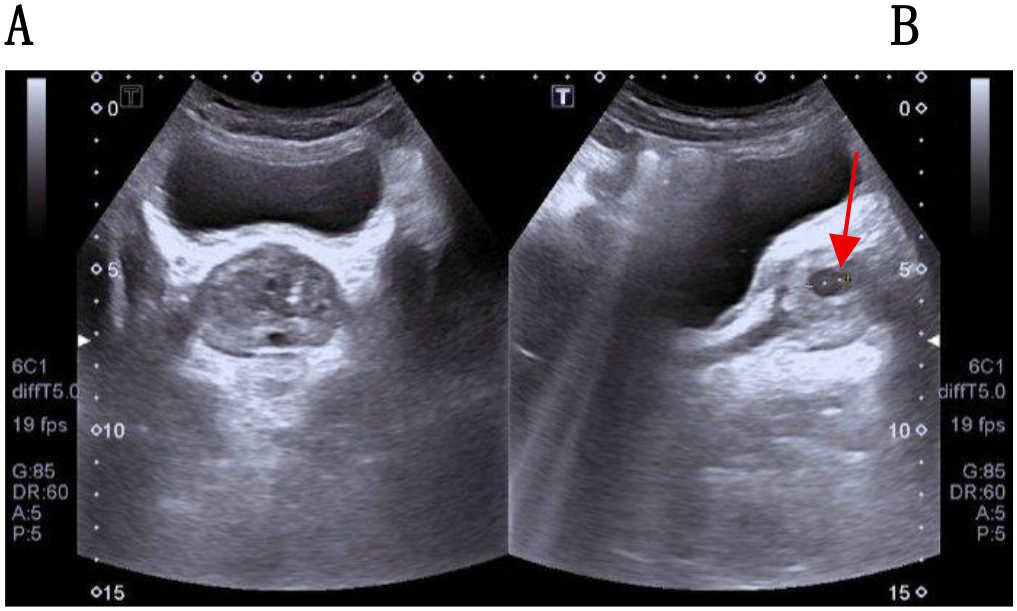
The patient’s urinary system ultrasound images. **(A)** The inner gland of the prostate is enlarged, and multiple hyperechoic foci are visible in the inner gland. **(B)** A relatively large cystic echo is seen within the prostate gland.

The urinary system ultrasound (**Figure 1**) revealed a solid nodule within the prostate. The prostate exhibited a full appearance with dimensions approximately 48×38×40 mm and a volume of 37.3 cm³. The inner gland was enlarged with an increased inner-to-outer gland ratio. Multiple hyperechoic foci were visible within the inner gland, along with multiple cystic echoes within the glandular tissue, the largest measuring 6.4 mm. No significant nodular shadows were observed. Prostate magnetic resonance imaging (MRI) revealed a nodule with abnormal signal intensity in the right mid-transition zone, suggestive of prostate cancer (Prostate Imaging Reporting and Data System, PI-RADS 4). The prostatic capsule appeared intact, and no enlarged lymph nodes were observed in the bilateral pelvic walls or inguinal regions. However, the patient’s serum prostate-specific antigen (PSA) level was 0.929 ng/mL (normal range: <4.0 ng/mL), falling within the normal range. The Free Prostate-Specific Antigen/Total Prostate-Specific Antigen (FPSA/TPSA) ratio was elevated at 0.34. Other routine hematologic and biochemical results showed no significant abnormalities.

**Figure 2:**
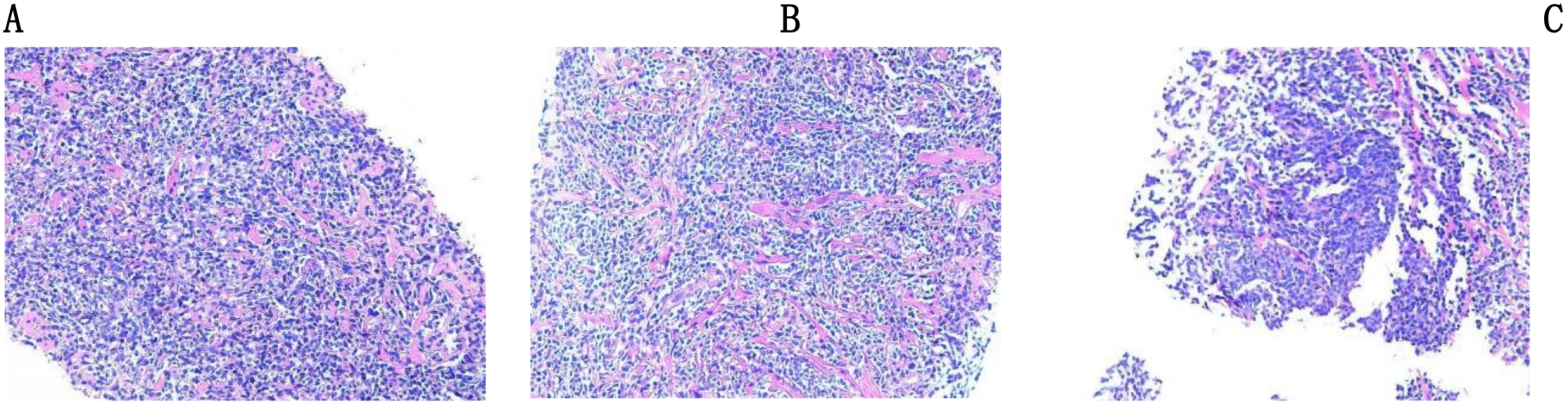
Results of prostate needle biopsy. **(A)** Diffusely distributed tumor cells with moderately sized cell nuclei, fine chromatin, and inconspicuous nucleoli; lymphoepithelial lesions are observed. **(B)** Increased pink fibrous connective tissue. The tumor cell nuclei have fine chromatin and relatively uniform morphology, predominantly composed of marginal zone B cells; prominent lymphoepithelial lesions are visible. **(C)** Tumor cells showing diffuse aggregation, with fine chromatin in their nuclei and unremarkable nucleoli.

Subsequently, an ultrasound-guided prostate biopsy was performed on the patient, revealing (**Figure 2**): (Prostate Segments 1, 2, 3, 5, 6) Lymphoid tissue hyperplastic lesion. (Prostate Segments 4, 7-12) Benign prostatic hyperplasia. Immunohistochemical staining showed positivity for CD20, CD79a, CD23, CD21, and BCL-2 antibodies; partial positivity for CD45 and CD43 antibodies; and negativity for PSA, NKX3.1, CKP, P504S, CD3, CD5, BCL-6, Cyclin D1, CD10, MUM1, C-myc, CgA, Syn, EBER, LEF-1, and negativity for SOX-11. Gene rearrangement analysis demonstrated a monoclonal B-cell population. Combined with the immunohistochemistry and molecular testing (Ig gene rearrangement) results, the findings were highly suggestive of MALT lymphoma. Bone marrow aspiration revealed granulocytopenia, indicating no bone marrow involvement.

PET-CT imaging revealed focal Fluorodeoxyglucose (FDG) metabolic enhancement in the right transitional zone of the prostate, with no evidence of lymphoma involvement in other regions. Whole-body bone scan also showed no metastatic lesions. Gastroscopy and colonoscopy results demonstrated no significant abnormalities. Based on the comprehensive evaluation of these examinations and pathological findings, the patient is considered to have prostate MALT lymphoma, Ann Arbor stage IE.

The patient declined the chemotherapy plus rituximab regimen and opted for external beam radiotherapy to the prostate. Detailed radiotherapy planning was as follows: (1) Simulation and Localization: The patient was positioned supine and immobilized with a thermoplastic shell. CT simulation was performed with a slice thickness of 5 mm. (2) Target Volume Definition: The clinical target volume (CTV) encompassed the radiologically visible prostate and seminal vesicles. The planning target volume (PTV) was generated by a three-dimensional uniform 5-mm expansion from the CTV. (3) Treatment Technique: Treatment was delivered using a linear accelerator with 6 MV X-rays and a multi-field dynamic intensity-modulated radiotherapy (IMRT) technique. The treatment was administered in one cycle over 3 weeks (5 fractions per week), with a total dose of 30.0 Gy in 15 fractions of 2.0 Gy each. (4) Organ-at-Risk (OAR) Constraints: Plan verification demonstrated that the doses received by the rectum, bladder, bilateral femoral heads, and small bowel were all within the tolerable dose range. (5) Dose Distribution: The pelvic IMRT plan achieved highly conformal dose distribution to the target. 95% of the PTV received the prescription dose, effectively reducing the radiation dose to surrounding normal tissues.

During radiotherapy, the patient developed only Grade 1 myelosuppression (white blood cell count 3.1×10^9^/L, neutrophil count 1.63×10^9^/L) without fever or infection. The condition normalized after treatment with subcutaneous human granulocyte colony-stimulating factor. No other adverse events of Grade 2 or higher were observed.

Subsequently, the patient attended follow-up outpatient visits at 1 week, 1 month, and 4 months after completing radiotherapy. Abdominal and urinary tract ultrasound examinations at these time points showed significant tumor regression with no signs of enlargement or recurrence. Furthermore, the patient’s urinary obstructive symptoms were completely relieved.

## 3 Discussion

Primary prostate lymphoma is extremely rare clinically, accounting for merely 0.09% of prostate tumors and 0.1% of all lymphomas (1). Diffuse large B-cell lymphoma is the most commonly reported histological subtype in the literature, whereas the MALT lymphoma reported herein is relatively uncommon. Currently, internationally recognized diagnostic criteria for primary prostate lymphoma include: (1) Tumor confined to the prostate and adjacent soft tissues; (2) No evidence of peripheral lymph node involvement; (3) No systemic lymphoma detected within at least one month after primary tumor diagnosis (2). In this case, the patient’s PET-CT indicated that the lesion was confined to the transitional zone of the prostate. MRI showed no evidence of extracapsular extension or regional lymph node metastasis. ECT and gastrointestinal endoscopy revealed no lymphoma involvement at other sites. Bone marrow aspiration did not indicate bone marrow involvement, and no systemic lymphoma manifestations were observed during follow-up, which is consistent with the diagnostic criteria for primary prostate lymphoma. Furthermore, the absence of systemic clinical symptoms and imaging evidence makes secondary disease or systemic lymphoma highly unlikely. According to the Ann Arbor staging system for lymphoma, this case can be diagnosed as stage IE. Accurate staging is essential for formulating the subsequent treatment plan.

In reported cases, most patients present with normal PSA levels, with only a minority showing elevation. In clinical practice, a prostate MRI lesion with a PI-RADS score of 4 typically indicates a high risk of prostate carcinoma and is often associated with elevated PSA. However, this patient had normal PSA levels. Since lymphoma is a tumor of lymphoid origin that does not secrete PSA, the possibility of prostate lymphoma, rather than solely prostate carcinoma, should be considered. The differentiation between prostatic lymphoma and prostate carcinoma relies primarily on pathological and immunohistochemical findings. Prostatic lymphoma is a tumor of lymphoid origin, characterized by diffuse lymphocytic infiltration, with its main immunohistochemical markers being positivity for CD45 and CD20. In contrast, prostate carcinoma is an epithelial tumor, predominantly exhibiting acinar or ductal structures, and its principal immunohistochemical markers are positivity for CKP and PSA.

An immunohistochemical profile showing CD45 positivity and CKP negativity effectively excludes an epithelial origin of the tumor. In this patient, the findings of CD45 positivity and CKP negativity strongly indicate a tumor of lymphoid origin. Further subtyping can be achieved through B- and T-cell lineage markers.

The definitive subtyping of prostate lymphoma relies primarily on immunohistochemistry and molecular testing results. In this case, immunohistochemistry was positive for core B-cell markers (CD20, CD79a) and markers typical of MALT lymphoma (CD21, CD23, BCL-2), with partial positivity for CD43. It was negative for markers of other lymphoma subtypes, allowing for differential diagnosis: T-cell lymphoma (CD3); follicular lymphoma (CD10, BCL-6); mantle cell lymphoma (characteristic markers Cyclin D1, SOX-11); diffuse large B-cell lymphoma (MUM1, CD30); chronic lymphocytic leukemia/small lymphocytic lymphoma (LEF-1, CD5, EBER); and neuroendocrine markers (CgA, Syn). The negativity for these key markers of other subtypes, combined with the monoclonal B-cell population indicated by molecular testing, confirms the diagnosis of MALT lymphoma.

Owing to the paucity of reported cases, no standardized therapeutic protocol has been established for prostate lymphoma to date for prostate lymphoma. Treatment approaches documented in the literature include radical prostatectomy, radical radiotherapy, chemotherapy, or chemotherapy combined with radiotherapy (3), with R-CHOP chemotherapy (rituximab + cyclophosphamide + doxorubicin + vincristine + prednisone) (with or without radiotherapy) being the preferred clinical regimen (4). As a systemic treatment, the toxicities associated with chemotherapy include myelosuppression, nausea, and vomiting. Specifically, doxorubicin can induce cardiotoxicity, while vincristine is associated with neurotoxicity. Furthermore, patients face an increased risk of infection (5).

The core value of surgical interventions (such as radical prostatectomy or transurethral resection of the prostate [TURP]) lies in two aspects: first, they can significantly improve acute urinary obstruction symptoms in patients, rapidly alleviating urgent conditions like urinary retention; second, when the pathology results from needle biopsy are inconclusive, fresh pathological tissue obtained during surgery can further clarify the diagnosis, providing a basis for subsequent treatment. However, it should be noted that there is currently insufficient evidence to demonstrate that surgery improves overall patient survival rates (6). The perioperative risks of surgical intervention include hemorrhage, infection, urinary incontinence, and erectile dysfunction.

Reviewing clinical cases over the past five years reveals a clear trend in treatment selection: most patients received R-CHOP chemotherapy (with or without radiotherapy), followed by surgical intervention. Notably, patients who did not undergo adjuvant chemoradiotherapy after surgery exhibited significantly increased risks of tumor recurrence and mortality (7, 8), underscoring the critical importance of postoperative adjuvant chemoradiotherapy in controlling disease progression.

Given the patient’s early tumor stage, advanced age, comorbidities (hypertension and diabetes mellitus), and limited physical tolerance—combined with the patient’s refusal of the chemotherapy plus rituximab regimen—the clinical decision was made to proceed with radical radiotherapy as the sole treatment modality. Radiotherapy alone is relatively less commonly used in the treatment of primary prostate lymphoma, yet its advantages remain prominent: on one hand, it effectively alleviates acute urinary obstruction symptoms; on the other, it reduces the local tumor recurrence rate (2). Compared to chemotherapy, radiotherapy not only offers a shorter treatment cycle but also achieves significant tumor regression in the later stages (with efficacy comparable to surgery), while causing less trauma to patients. Considering that most patients with primary prostate lymphoma are elderly males with relatively weaker physical tolerance, the low-trauma nature of radiotherapy makes it significantly more acceptable to these patients. Following radiotherapy, the patient achieved complete resolution of urinary obstruction symptoms without experiencing any severe adverse reactions throughout the treatment course. Short-term follow-up at 4 months demonstrated significant tumor regression with no evidence of recurrence, thereby preliminarily validating both the safety and short-term efficacy of the treatment. Notably, primary prostatic MALT lymphoma, a relatively indolent subtype of NHL, exhibits slow disease progression, and the assessment of its long-term recurrence risk necessitates long-term follow-up and monitoring. A major limitation of this case report, however, is the relatively short follow-up duration (only 4 months), which fails to validate the long-term efficacy of radical radiotherapy. Furthermore, neither the long-term recurrence risk of the disease nor the treatment-related late adverse reactions can be effectively evaluated.

## 4 Conclusion

The clinical symptoms of primary prostatic lymphoma lack specificity, and its imaging findings are often indistinguishable from those of prostate carcinoma. Therefore, definitive diagnosis relies on the pathological results of a prostate biopsy. Should the biopsy findings prove inconclusive—for instance, due to insufficient specimen volume or inadequate diagnostic evidence—pathological examination of a surgically resected specimen may be necessary for confirmation.

In clinical practice, when managing patients who present with lower urinary tract symptoms in the presence of normal or only mildly elevated PSA levels, the possibility of primary prostatic lymphoma should be considered. A markedly elevated prostate-specific antigen (PSA) level is not a typical feature of this disease; thus, this characteristic can serve as a key diagnostic clue for differentiation from prostate carcinoma.

It is essential to emphasize that establishing an early and accurate diagnosis is crucial for improving the prognosis of patients with primary prostatic lymphoma. Consequently, for patients with early-stage, localized disease, those with poor tolerance for systemic therapy, or those who decline chemotherapy, radiotherapy with curative intent may be considered as a potential individualized treatment option. However, it cannot yet be regarded as a universally applicable standard regimen. Its long-term efficacy and safety require further validation through larger case series and long-term follow-up data.

## Data Availability

The original contributions presented in the study are included in the article/supplementary material. Further inquiries can be directed to the corresponding author.
